# UK Medical Cannabis Registry palliative care patients cohort: initial experience and outcomes

**DOI:** 10.1186/s42238-021-00114-9

**Published:** 2022-01-04

**Authors:** Devaki Nimalan, Michal Kawka, Simon Erridge, Mehmet Ergisi, Michael Harris, Oliver Salazar, Rayyan Ali, Katerina Loupasaki, Carl Holvey, Ross Coomber, Michael Platt, James J. Rucker, Shaheen Khan, Mikael H. Sodergren

**Affiliations:** 1 Department of Surgery and Cancer, St Mary’s Hospital, Imperial College Medical Cannabis Research Group, Imperial College London, Academic Surgical Unit, 10th Floor QEQM, South Wharf Road, London, W2 1NY UK; 2 Sapphire Medical Clinics, London, UK; 3 St. George’s Hospital NHS Trust, London, UK; 4 Department of Psychological Medicine, Kings College London, London, UK; 5 South London & Maudsley NHS Foundation Trust, London, UK; 6 Guy’s & St. Thomas’ NHS Foundation Trust, London, UK

**Keywords:** Cannabinoids, Medical cannabis, Health-related quality-of-life, Palliative care

## Abstract

**Introduction:**

Palliative care aims to improve quality of life through optimal symptom control and pain management**.** Cannabis-based medicinal products (CBMPs) have a proven role in the treatment of chemotherapy-induced nausea and vomiting. However, there is a paucity of high-quality evidence with regards to the optimal therapeutic regimen, safety, and effectiveness of CBMPs in palliative care, as existing clinical trials are limited by methodological heterogeneity. The aim of this study is to summarise the outcomes of the initial subgroup of patients from the UK Medical Cannabis Registry who were prescribed CBMPs for a primary indication of palliative care, cancer pain and chemotherapy-induced nausea and vomiting, including effects on health-related quality of life and clinical safety.

**Methods:**

A case series from the UK Medical Cannabis Registry of patients, who were receiving CBMPs for the indication of palliative care was undertaken. The primary outcome consisted of changes in patient-reported outcome measures including EQ-5D-5L, General Anxiety Disorder-7 (GAD-7), Single-Item Sleep Quality Scale (SQS), Pain Visual Analog Scale (VAS) and the Australia-Modified Karnofsky Performance Scale at 1 and 3 months compared to baseline. Secondary outcomes included the incidence and characteristics of adverse events. Statistical significance was defined by *p*-value< 0.050.

**Results:**

Sixteen patients were included in the analysis, with a mean age of 63.25 years. Patients were predominantly prescribed CBMPs for cancer-related palliative care (*n* = 15, 94%). The median initial CBD and THC daily doses were 32.0 mg (Range: 20.0–384.0 mg) and 1.3 mg (Range: 1.0–16.0 mg) respectively. Improvements in patient reported health outcomes were observed according to SQS, EQ-5D-5L mobility, pain and discomfort, and anxiety and depression subdomains, EQ-5D-5L index, EQ-VAS and Pain VAS validated scales at both 1-month and 3-months, however, the changes were not statistically significant. Three adverse events (18.75%) were reported, all of which were either mild or moderate in severity.

**Conclusion:**

This small study provides an exploratory analysis of the role of CBMPs in palliative care in the first cohort of patients since CBMPs legalisation in the UK. CBMPs were tolerated with few adverse events, all of which were mild or moderate and resolved spontaneously. Further long-term safety and efficacy studies involving larger cohorts are needed to establish CBMPs role in palliative care, including comparisons with standard treatments.

## Introduction

Achieving optimal pain and symptom control is a crucial component of palliative and end-of-life care. There is a pertinent need for the provision of effective treatment with up to 40 million people requiring palliative care globally each year (World Health Organization, 2020). Furthermore, evidence suggests that the pain incurred by life-limiting illnesses, particularly cancer, remains largely under-treated (Breivik et al., 2009).

A growing body of literature suggests that cannabinoids, terpenes, and flavonoids exert widespread effects on neurotransmission, neuroendocrine signalling, and inflammatory processes (Huang et al., 2016). This potentially makes cannabis-based medicinal products (CBMPs) an emerging multi-faceted therapeutic option in managing primary chronic pain, cancer pain and neuropathic pain (Romero-Sandoval et al., 2018). CBMPs, generally refer to pharmaceuticals and non-approved compounds which interact with the endocannabinoid system. However, they can also refer to cannabinoid-based medicines, endocannabinoid system modulators or cannabinoid receptor modulators, in light of lack of consensus of nomenclature. Possible benefits have also been shown for some neurological and psychiatric conditions, such as anxiety-predominant disorders. Combined, these effects make the use of CBMPs as part of end-of-life care for patients a promising yet relatively unexplored avenue.

There is a paucity of evidence to guide best prescribing practices and optimal therapeutic regimes. Whilst randomised control trials (RCTs) constitute the highest quality evidence, current research remains limited in providing CBMP patient data. Formalised patient registries can be the source of high-quality, naturalistic observational data to answer these questions until RCTs are conducted. We therefore describe a preliminary exploratory analysis of outcomes of patients from the UK Medical Cannabis Registry who were treated with CBMPs for diagnoses related to palliative care. We aimed to investigate therapeutic formulations, adverse events incidence and character, and patient-reported outcome measures pertaining to the quality of life.

## Methods

A case series of the initial participants of the UK Medical Cannabis Registry treated with CBMPs for indications of palliative care, cancer pain and chemotherapy-induced nausea and vomiting was undertaken. Palliative care was defined as treatments for physical and psychological symptoms caused by life-threatening or life-limiting conditions. Participants who had recorded PROMs at baseline with at least 1 follow-up date (1 and/or 3 months) were included in the study (*n* = 16).

The UK Medical Cannabis Registry is the first registry in the UK that collates prospective longitudinal clinical data from patients treated with CBMPs. It was set up in 2019, and captures patients treated within the United Kingdom and outside of the National Health Service (NHS). The UK Medical Cannabis Registry is privately owned and managed by Sapphire Medical Clinics. Clinicopathological information, comorbidities, drug and alcohol history and medication details are inputted into the registry prospectively by clinical staff. Data on cannabis use status was also collected, and for those who had previously or were presently taking non-prescription cannabis, a novel metric of “gram years” was calculated as previously described by our group (Erridge et al., 2021). Pseudonymised clinical data on patient-reported outcome measures (PROMs) and adverse events were collected.

All participants completed five validated quality of life PROMs, including the EQ-5D-5L (Herdman et al., 2011), a health status measure assessing quality of life amongst 5 domains (mobility, self-care, usual activities, pain/discomfort, and anxiety/depression) with 5 levels of severity (no problems, slight problems, moderate problems, severe problems, and extreme problems), General Anxiety Disorder-7 (GAD-7) (Löwe et al., 2008), Single-Item Sleep Quality Scale (SQS) (Snyder et al., 2018), the Australia-Modified Karnofsky Performance Scale (AKPS) (Abernethy et al., 2005) and the Pain Visual Analog Scale (VAS), a unidimensional measure of pain intensity anchored by “0 – no pain at all” and “10 – pain as bad as it could be” (Hawker et al., 2011). Adverse events were recorded according to the Common Terminology Criteria for Adverse Events (CTCAE v4.0).

PROMs data were compared from baseline to 1- and 3-month follow-ups. Paired matched Wilcoxon rank sum tests were utilised for non-parametric data sets and paired t-test were used for parametric data sets, as determined by Shapiro-Wilks normality tests. Statistical significance was defined as *p*-value < 0.050. All statistical analyses were conducted using Statistical Package for Social Sciences (SPSS) version 27.0.

## Results

Sixteen patients were included in the analysis. The mean age of participants was 63.25 ± 12.27 years, with equal sex distribution. Half of the participants (*n* = 8, 50%) had never used cannabis previously, 4 (25%) were current cannabis users, and 4 (25%) were ex-users. Amongst the current users, gram-years ranged from 1 to 10, with the mean of 2.4; amongst ex-users gram-years ranged from 0.1 to 1, with an average of 0.35. Amongst current users, the average daily THC consumption (estimated based on 18–20% THC content in cannabis) was 20–400 mg, with an average of 120 mg per day. 6 (37.5%) participants died during the study period.

The most common primary diagnosis was palliative care (*n* = 12, 75%), followed by cancer pain (*n* = 3, 18.75%) and chemotherapy-induced nausea and vomiting (*n* = 1, 6.25%).

Most participants (*n* = 14, 87.5%) were prescribed two different CBMPs, all of which were oil preparations. The median initial CBD dose was 32.0 mg (Range: 20.0–384.0 mg). The median initial THC dose was 1.3 mg (Range: 1.0–16.0 mg). The most commonly used preparations were 50 mg/ml CBD oil (Adven 50, Curaleaf International) and 20 mg/ml THC oil (Adven 20, Curaleaf International).

In total, there were three adverse events (*n* = 3, 8.75%). Adverse events experienced by participants included lethargy (*n* = 1, 6.25%), ataxia (*n* = 1, 6.25%) and dysgeusia (*n* = 1, 6.25%). Of these, 2 (12.5%) were reported as mild and 1 (6.25%) was reported as moderate. When analysed based on the cannabis use status, ex-users, current users and those who never used cannabis had equal adverse event rates, with one event in each subgroup.

Pain VAS was initially reported as ‘severe’ (6.5 ± 2.07) however reduced to ‘mild to moderate’ (4.24 ± 2.91) at 1 month, and to ‘mild’ (1.00 ± 1.41) at 3 months. Mean SQS scores improved from 4.89 (± 2.32) at baseline to 6.89 (± 2.03) at 1 month, and 5.25 (± 3.02) at baseline to 7.75 (± 1.71) at 3 months. Mean SQS scores improved by 40.9% from baseline to 1 month, and 46.7% from baseline to 3 months. Overall, there were no significant improvements in mean SQS, median EQ-5D-5L Mobility, EQ-5D-5L Pain and Discomfort, EQ-5D-5L Anxiety and Depression, EQ-5D-5L Index, EQ-VAS and Pain VAS at 1-month and 3-month, when compared to the baseline (*p* > 0.05).

## Discussion

CBMPs were well tolerated with few adverse events, all of which were mild to moderate in severity and resolved spontaneously. No significant improvements were found in any of the outcome measures due to the small sample size of the study (*n* = 16). A post-hoc power calculation using data from this case series determined a sample size of 56 would be required to determine a significant difference at 3 months in reported EQ-5D-5L index values. An updated analysis shall be performed when this is available.

## Conclusion

This preliminary exploratory study provides an initial analysis of the role of CBMPs in palliative care in the first reported cohort of patients since the legalisation of CBMPs in the United Kingdom. CBMP treatment was well-tolerated with few adverse events, which were all mild to moderate in severity and resolved spontaneously. This data provides an insight into the safety and outcomes of CBMP treatment amongst palliative care patients and may help guide future clinical studies and prescribing practice. Further long-term safety and efficacy studies involving larger cohorts are needed to evaluate long-term prescribing outcomes, with comparisons with placebo and standard treatments for palliative symptom control.

## Data Availability

Data that support the findings of this study are available from the UK Medical Cannabis Registry. Restrictions apply to the availability of these data. Data specifications and applications are available from the corresponding author.

## Abbreviations

CBMPs: Cannabis-based medicinal products
GAD-7: General Anxiety Disorder-7
VAS: Visual Analog Scale
AKPS: Australia-Modified Karnofsky Performance Scale
PROMs: Patient-Reported Outcome Measures
CTCAE: Common Terminology Criteria for Adverse Events
SPSS: Statistical Package for Social Sciences
